# Anatomic vs. Acquired Image Frame Discordance in Spectral Domain Optical Coherence Tomography Minimum Rim Measurements

**DOI:** 10.1371/journal.pone.0092225

**Published:** 2014-03-18

**Authors:** Lin He, Ruojin Ren, Hongli Yang, Christy Hardin, Luke Reyes, Juan Reynaud, Stuart K. Gardiner, Brad Fortune, Shaban Demirel, Claude F. Burgoyne

**Affiliations:** 1 Optic Nerve Head Research Laboratory, Legacy Health, Portland, Oregon, United States of America; 2 Discoveries in Sight Research Laboratories of the Devers Eye Institute, Legacy Health, Portland, Oregon, United States of America; Medical Faculty Mannheim of the University of Heidelberg, Germany

## Abstract

**Purpose:**

To quantify the effects of using the fovea to Bruch's membrane opening (FoBMO) axis as the nasal-temporal midline for 30° sectoral (clock-hour) spectral domain optical coherence tomography (SDOCT) optic nerve head (ONH) minimum rim width (MRW) and area (MRA) calculations.

**Methods:**

The internal limiting membrane and BMO were delineated within 24 radial ONH B-scans in 222 eyes of 222 participants with ocular hypertension and glaucoma. For each eye the fovea was marked within the infrared reflectance image, the FoBMO angle (θ) relative to the acquired image frame (AIF) horizontal was calculated, the ONH was divided into 30°sectors using a FoBMO or AIF nasal/temporal axis, and SDOCT MRW and MRA were quantified within each FoBMO vs. AIF sector. For each sector, focal rim loss was calculated as the MRW and MRA gradients (i.e. the difference between the value for that sector and the one clockwise to it divided by 30°). Sectoral FoBMO vs. AIF discordance was calculated as the difference between the FoBMO and AIF values for each sector. Generalized estimating equations were used to predict the eyes and sectors of maximum FoBMO vs. AIF discordance.

**Results:**

The mean FoBMO angle was −6.6±4.2° (range: −17° to +7°). FoBMO vs. AIF discordance in sectoral mean MRW and MRA was significant for 7 of 12 and 6 of 12 sectors, respectively (p<0.05, Wilcoxon test, Bonferroni correction). Eye-specific, FoBMO vs. AIF sectoral discordance was predicted by sectoral rim gradient (p<0.001) and FoBMO angle (p<0.001) and achieved maximum values of 83% for MRW and 101% for MRA.

**Conclusions:**

Using the FoBMO axis as the nasal-temporal axis to regionalize the ONH rather than a line parallel to the AIF horizontal axis significantly influences clock-hour SDOCT rim values. This effect is greatest in eyes with large FoBMO angles and sectors with focal rim loss.

## Introduction

Since the introduction of the concept of the ‘cup/disc ratio’ by Armaly in the 1960s [1], clinical disc examination has required the identification of the outer and inner borders of the neuroretinal rim, (the optic disc margin and optic disc cup, respectively). The amount of rim tissue is then estimated within the apparent plane of the disc margin as either the rim width (expressed in mm) or as the ratio of the size of the cup to the size of the disc (expressed as a cup/disc ratio using either diameter or area measurements) [1] or as rim area (expressed in mm^2^) [2], [3]. Rim measurements are regionalized relative to a nasal temporal axis that is assumed to be parallel to the horizontal axis of the acquired image frame (AIF), whether the image frame is acquired through fundus imaging or slit lamp biomicroscopy, without regard to the anatomic relationship between the optic nerve head (ONH) and the fovea.

**Figure 1:**
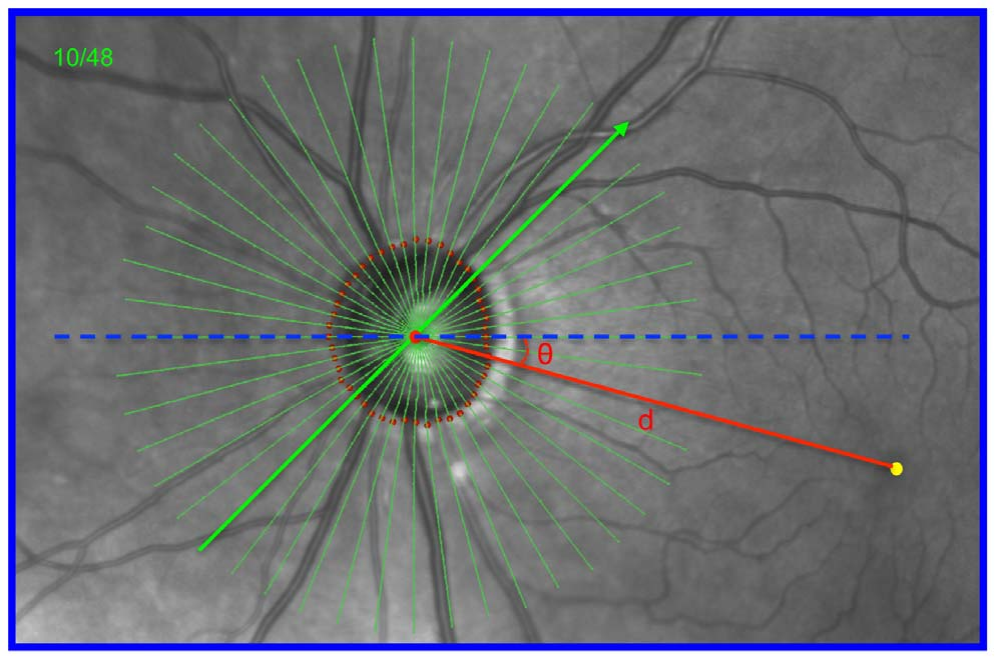
Identification of the fovea-to-BMO centroid (FoBMO) axis (red) relative to the Acquired Image Frame (AIF- blue outline) horizontal axis (dashed blue line), the FoBMO angle (θ) and the FoBMO distance (d) on the infrared (IR) fundus image of a representative study eye (DIS368). In each study eye, the FoBMO axis, the FoBMO angle and the FoBMO distance were digitally generated relative to the SDOCT ONH data set through the following steps. 1) The fovea (yellow dot) was digitally identified on the IR image by one clinician (CFB). 2) The delineated BMO points from 24 of the 48 acquired SDOCT B-scans were projected onto the IR image plane allowing the geometric center (BMO centroid - large red dot) to be located relative to the acquired SDOCT data. 3) The FoBMO axis was defined to be the line (red) connecting the fovea and BMO centroid. 4) The FoBMO angle (θ) was defined to be the angle between the FoBMO axis and the AIF horizontal axis (blue dashed line). The FoBMO distance (d) was defined to be the distance between the BMO centroid and the assigned fovea. The FoBMO angle is −16° and the FoBMO distance is 4.3 mm in this eye (see Figure 4).

Recent spectral domain optical coherence tomography (SDOCT) findings call into question the current concepts of the clinical disc margin, rim quantification and its regionalization from both anatomic [4] and geometric [5]–[8] perspectives. Based on these findings, and previous work by other groups on retinal anatomy and its relationship to structure-function correlation [9]–[14], we have argued for making minimum rim width (MRW) and area (MRA) measurements relative to SDOCT Bruch's Membrane Opening (BMO) [8], [15]–[17] and linking ONH neuroretinal rim as well as peripapillary and macular retinal nerve fiber layer (RNFL) regionalization to the axis between the centroid of BMO and the fovea, which we term the foveal-BMO or FoBMO axis (Figure 1) [18].

In clinical fundus images, the fovea is located below the level of the ONH (relative to the horizontal axis of the AIF) in most individuals (Figure 1). In a series of previous studies, the mean angle between the fovea and the center of the ONH was most commonly −6 to −7° (the fovea being 6–7° below, causing the blind spot to be below the horizontal midline during functional testing), with a range between −17° (below) and +7° (above) (reviewed in [18]). The position of these two structures relative to the AIF may vary by as much as 6.4±3.8° within images of the same eye obtained on the same imaging day due to cyclotorsion and head tilt [14]. Their position also varies considerably between subjects due to differences in retinal anatomy. However, because the anatomic path of the RNFL axon bundles between the fovea and the ONH is organized relative to the FoBMO axis [9], [19], [20], it is an anatomically consistent landmark for the regionalization of the ONH and retinal tissues in all human eyes.

**Figure 2:**
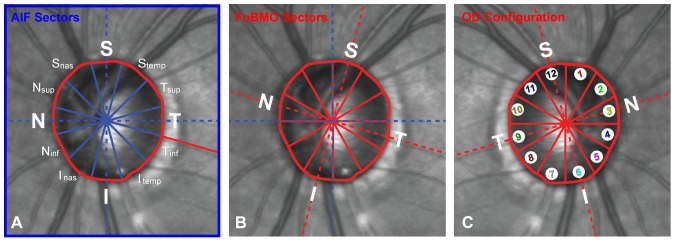
AIF versus FoBMO 30° ONH regionalization of a representative study eye (DIS368). This is the same eye (DIS368) as shown in Figure 1. (A) AIF (blue outline) regionalization with the naming convention for the twelve 30° sectors. (B) FoBMO regionalization of the same eye. Note that for this eye the actual anatomy being measured within the Superior (S) sector (as an example) is different within the AIF (A) compared to the FoBMO (B) regions. (C) FoBMO 30° sectors from the left eye shown in panel B, flipped into OD configuration and numbered with separate colors in the manner in which the data for all 222 eyes are presented in Figure 9.

Current clinical examination, image acquisition and data analysis algorithms assume that neuroretinal rim width and area in a given sector refer to approximately the same anatomic location in all human eyes. However, this assumption is only true when the ONH of each individual eye is regionalized using the FoBMO axis as the Nasal/Temporal horizontal midline (Figures 1 and 2). Within an individual eye, as the FoBMO angle increases relative to the AIF horizontal axis, the anatomic discordance (the difference in the anatomic location being measured) of an individual sector using AIF vs. FoBMO regionalization may be large (Figure 2). Because AIF sector positions can refer to measurements from different anatomic locations in different eyes, artificially large inter-individual differences in sectoral neuroretinal rim width and area in normative databases may be introduced by AIF regionalization. As a result, the limits of normal variation in these measurements are likely increased, which may decrease their diagnostic accuracy.

One of the hallmarks of glaucomatous neuroretinal rim loss is that it can be focal or sectoral in character, leading to sectoral rim thickness changes within a damaged eye that are more abrupt than sectoral rim thickness variation within normal ONHs [21]. We propose the term sectoral rim gradient to refer to the magnitude of focal rim loss calculated as the difference in rim anatomy between a sector and its neighboring sectors within an individual eye. To quantify this phenomenon by sector, we introduce the parameters *minimum rim width gradient* and *minimum rim area gradient* which are related to but different from previously described strategies for quantifying retinal nerve fiber layer thickness RNFLT [22], [23] and visual field sensitivity gradients [24].

The purpose of the present study was to quantify the eye-specific effects of using FoBMO versus AIF regionalization of SDOCT ONH neuroretinal rim data from 222 eyes of 222 participants with high-risk ocular hypertension and glaucoma enrolled in the Portland Progression Project (P3 study). Our hypothesis was that a combination of FoBMO angle and rim gradient would predict the sectors (and eyes) with greatest FoBMO vs. AIF rim discordance. Our data suggest that the frequency and magnitude of FoBMO vs. AIF sectoral SDOCT MRW and MRA discordance are substantial and greatest within sectors that display elevated rim gradients (i.e. focal rim loss) from eyes with the largest FoBMO axis.

## Methods

All data were obtained from an ongoing longitudinal study, the Portland Progression Project (P3 study) [25]. The P3 study, funded by the National Institutes of Health, is an ongoing longitudinal study of the course and risk factors for glaucomatous progression. Participants with high-risk ocular hypertension and glaucoma (defined below) have been recruited prospectively from the Devers Eye Institute, or other ophthalmic practices in the Portland, OR metropolitan area as previously described [26], [27]. At the time of recruitment, all participants were fully informed of the potential risks and benefits of the study and provided their voluntary written consent. All P3 study procedures follow the tenets of the Declaration of Helsinki, are in accordance with the Health Insurance Portability and Accountability Act and were approved by the Institutional Review Board at Legacy Health.

At study entry, participants either had early glaucoma with visual field loss less severe than −6 decibels (dB) for mean deviation (MD) or ocular hypertension (untreated IOP greater than 22 mm Hg) plus one or more risk factors for glaucoma as determined by their clinician. Risk factors for ocular hypertension included: age >70, systemic hypertension, migraine, diet-controlled diabetes, peripheral vasospasm, African ancestry, self-reported family history of glaucoma and suspicious optic nerve head appearance (cup-disc ratio asymmetry >0.2, neuroretinal rim notching or narrowing or disc hemorrhage). All participants also met the following criteria for both eyes: best corrected visual acuity of 20/40 or better and spectacle refraction < ±5.00-D sphere and < ±2.00-D cylinder [26], [27]. Potential participants were excluded if they had any other previous or current ocular or neurologic disease likely to affect the visual field and previous ocular surgery (except uncomplicated cataract surgery).

### SDOCT Imaging

Standard Spectralis 870 nm SDOCT (Heidelberg Engineering GmBH, Heidelberg, Germany) was used to image both eyes of all 222 patients. Forty-eight radial B-scans were acquired over a 15° area. Each B-scan consisted of 768 A-scans and was the average of 9 repetitions. All acquired SDOCT datasets had a quality score above 15. If both eyes in each patient met the above inclusion criteria, the eye with the better SDOCT image quality was selected for further quantification.

### SDOCT Delineation and Parameterization

**Figure 3:**
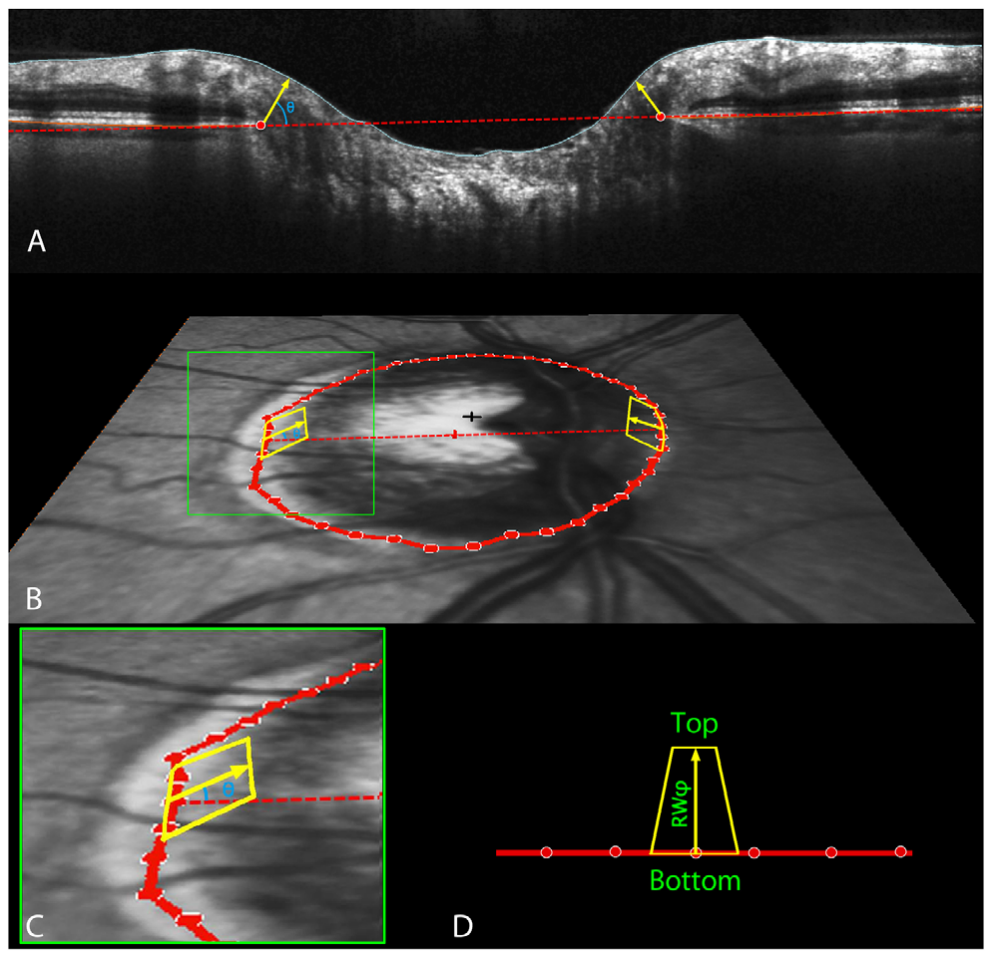
SDOCT ONH neuroretinal rim parameterization. A. Within each radial scan, Bruch's Membrane Opening (BMO) was delineated (red circles). The minimum rim width within that sector (MRW_Sec_) was calculated, by finding the shortest distance from the BMO to the ILM (yellow arrow in Panel A). Within each section, the measures were averaged across sectors to give the sector measure BMO-MRW. This measure occurs at angle θ above the BMO plane (blue in Panels A and B, zoomed-in view in Panel C). Sectoral minimum rim areas (yellow areas in panels B, C and D) were calculated as the area of a trapezium at angle φ above the BMO plane (shown for simplicity as φ equaling θ, though this may not be the case for a given sector). The height of this trapezium was set equal to the rim width at this angle, 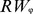. In the event that 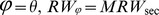. The base of the trapezium (bounded by the BMO) equaled the BMO circumference within that sector, 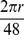, where *r* represents the distance from BMO centroid (red cross in Panel B) to the BMO point. The top of the trapezium (bounded by the ILM) was calculated from these accounting for the inclination angle φ, giving length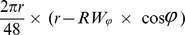. The area of each trapezium is calculated using the formula:
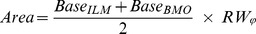
(Panel D).

The internal limiting membrane (ILM), RNFL, and BMO were delineated in 24 of the 48 B-scans (every other B-scan) by technicians masked to the clinical status, refractive error and FoBMO angle of the eye. Two SDOCT neuroretinal rim parameters were defined and quantified as previously described [8], [16]. BMO Minimum rim width (BMO-MRW) was defined to be the shortest distance from the BMO to the ILM within each B-scan (Figure 3A) [5]–[8], [15], [28]. The four sectoral measures within each 30° sector were averaged to give the sector measure for BMO-MRW. This measure occurs at an angle above the BMO plane (blue in Figure 3A and 3B, magnified view in Figure 3C). It should be noted that BMO-MRW is the minimum rim width measured from BMO, which is an anatomically consistent landmark in most SDOCT ONH B-scans. The actual minimum rim width at any location may occur deeper within the neural canal but cannot be consistently visualized[16].

Sectoral minimum rim areas (MRA, yellow areas in Figure 3B, 3C and 3D) [16] were calculated as the area of a trapezium at angle φ above the BMO plane (shown for simplicity as φ equaling θ, though this may not be the case for a given sector). The height of this trapezium was set equal to the rim width at this angle, 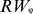. In the event that 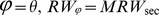. The long base of the trapezium (bounded by the BMO) equaled the BMO circumference within that sector, 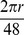, where r represents the distance from BMO centroid (red cross in Figure 3B) to the BMO point. The short base of the trapezium (bounded by the ILM) was calculated from these accounting for the inclination angle φ, giving length 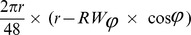. The area of each trapezium is calculated using the formula (Figure 3D): 
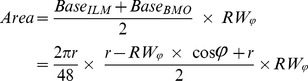



Both MRW and MRA were calculated on a 30° degree (12 clock-hours) sector basis.

### SDOCT ONH Regionalization

Separate AIF and FoBMO 30° regionalization of the SDOCT ONH rim data for each eye (Figure 2) were performed as follows.

### FoBMO Axis Identification (Figure 1)

For each eye in the study, one experienced clinician (CFB) identified the position of the fovea within the infrared Scanning Laser Ophthalmoscopy (SLO) reflectance image (IR image) acquired by the Spectralis as part of the SDOCT scan recording. The pixel closest to the point identified was recorded as the position of the fovea for that eye; 2) the delineated BMO points from 24 of the 48 acquired SDOCT B-scans were projected onto the IR image plane allowing the geometric center (BMO centroid) to be located; 3) the FoBMO axis was defined to be the line connecting the fovea and BMO centroid; 4) the FoBMO angle was defined to be the angle between the FoBMO axis and the AIF horizontal axis; and 5) the FoBMO distance was defined to be the distance between the BMO centroid and the assigned fovea.

### Intra- and Inter-delineator Reproducibility of FoBMO Axis Identification

Because the location of the fovea was retrospectively identified within an IR image rather than having been identified at the time of image acquisition on the basis of 3D information available from SDOCT scans (i.e. as the geometric center of the foveal pit [18]), intra- and inter-delineator reproducibility of this post hoc procedure was assessed as follows. Intra-delineator reproducibility was assessed by having the primary foveal delineator (CFB) separately identify the fovea in all 222 study eyes, 2 weeks after and masked to the first foveal delineation. Inter-delineator variability was assessed by having a second clinician (BF) separately delineate the fovea in all 222 study eyes. Intra-delineator variability was defined to be due to the difference between the primary delineator's two sets of foveal marks. Inter-delineator variability was defined to be due to the difference between the primary delineator's initial set of marks and the second delineator's marks.

#### FoBMO vs. AIF 30° Sectoral SDOCT Rim Tissue Regionalization (Figure 2)

Using the primary delineator's initial set of foveal marks, the SDOCT ONH rim data (MRW and MRA) for each eye were separately calculated for each 30° sector using either the AIF horizontal or the FoBMO axis as the Nasal-Temporal axis.

### FoBMO vs. AIF 30° Sectoral SDOCT Rim Discordance (Figure 2)

The magnitude of FoBMO vs. AIF discordance in the MRW and MRA measurements for each sector of each eye was calculated as the percent of the average of the two volumes, using the following formulas:
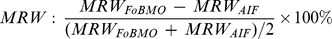





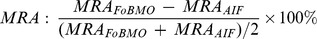



### Data Analysis

Intra- and inter-delineator limits-of-agreement were determined by Bland-Altman analysis for both FoBMO axis and distance [29]. Wilcoxon non-parametric t-tests were used to compare the rim values for all 222 eyes aligned to the FoBMO axis against the 222 rim values aligned to AIF for each 30° sector. The significance of each of the 12 t-tests was corrected for multiple comparisons using the Bonferroni method. The mean, 95% CI and range of sectoral, eye-specific discordance between FoBMO and AIF rim data for the 222 eyes were calculated. Percentage of eyes with a minimum of 1, 2 or 3 sectors in which the FoBMO vs. AIF rim data differed by greater than ±20% were identified.

### Quantifying Sectoral Neuroretinal Rim Gradient

To quantify sectoral rim gradient using both MRW and MRA, rim data for all eyes were converted to right eye orientation. A *one-sided* sectoral gradient for MRW and MRA was calculated (*MRW_FoBMO_*' and *MRA_FoBMO_*') for each sector as the difference in rim width between the value for that sector and the one clockwise to it, then divided by 30° for each sector. Using the superior sector as an example, 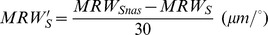. All analyses were performed using FoBMO sectoral regionalization of each study eye.

### Predicting FoBMO vs. AIF Discordance

A generalized estimating equation (GEE) model was applied to predict the magnitude of sectoral FoBMO versus AIF discordance, using two hypothesized factors: FoBMO angle (θ) and sectoral rim gradient while accounting for intra-eye inter-sector correlations. Interaction of the two factors was also taken account. The GEE model was implemented in RStudio (RStudio, Inc., Boston, MA) using the package ‘geepack’. Models were constructed separately using the FoBMO axis angle (θ), one-sided rim gradient (*MRW_FoBMO_*') and the interaction of FoBMO axis angle and one-sided rim gradient (*θ × MRW_FoBMO_*').

## Results

Table 1 summarizes the demographic information of the study participants. Two hundred and twenty-two eyes, of 222 individuals were included in the study, ranging from 33.7 to 89.8 years old (mean ± SD, 64.3±11.1). Mean IOP (± SD) on the day of imaging was 17.4±3.5 mmHg (range: 5.0 to 29.0 mmHg). Visual field MD (± SD) was −0.7±2.9 dB (range: −16.5 to 3.3 dB) while pattern standard deviation (PSD) (± SD) was 2.8±2.7 dB (range: 0.9 to 14.8 dB).

**Table 1:** Demographic and glaucoma-related characteristics of the cohort.

Parameter	Count/Mean ± SD
Number of eyes/participants	222/222
Eye: left/right (%)	51.3/48.7
Gender: M/F	92/130
Self-identified ethnicity (%):	
Caucasian	93.2
African descent	2.7
Asian descent	0.9
Hispanic descent	0.9
Native American	1.8
Others	0.5
Age (yrs)	64.3±11.1
Intraocular pressure (mmHg)	17.4±3.5
Mean deviation (dB)	−0.7±3.5
Pattern standard deviation (dB)	2.8±2.7
Central corneal thickness (μm)	557±40

### Distribution of FoBMO axis angle and distance (Figure 4)

**Figure 4:**
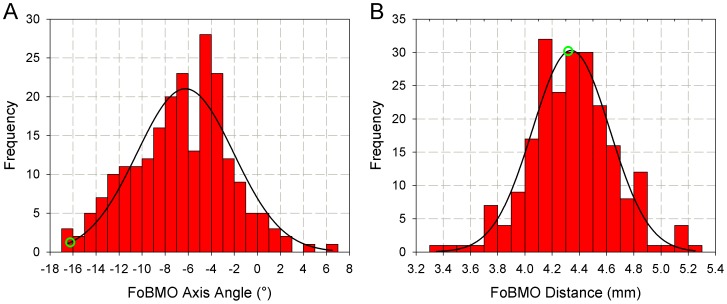
Histograms of FoBMO axis angle (A) and distance (B). FoBMO axis angle ranges from −17° to 7° (corresponding to “θ” in Figure 2A) while FoBMO distance ranges from 3.4 mm to 5.4 mm (corresponding to “d” in Figure 2B). The black lines are the Gaussian fits for the histograms. Shapiro-Wilk normality test finds p = 0.291 for FoBMO angle and p = 0.294, suggesting both histograms are normally distributed. The green circle represents the eye “DIS368” (Figures 1 and 2). The FoBMO Axis Angle data (panel A) appeared within a previous publication as part of a written communication (*Chauhan BC and Burgoyne CF (2013) From clinical examination of the optic disc to clinical assessment of the optic nerve head: a paradigm change. Am J Ophthalmol 156: 218-227 e212*.).

The FoBMO Axis Angle data in this report appeared within a previous publication [18] as part of a written communication. Histograms of the FoBMO axis angle (A) and distance (B) data for all 222 eyes are presented in Figure 4. FoBMO angle and distance data were normally distributed by Shapiro-Wilk normality test (p = 0.291 and p = 0.294, respectively). The FoBMO angle ranged from −17° to 7° (mean ± SD, −6.6°±4.2°) and the FoBMO distance ranged from 3378 μm to 5269 μm (mean ± SD: 4330±428 μm). Twelve of the 222 eyes (5.4%) demonstrated a positive FoBMO axis angle (fovea above the ONH) and 210 of the 222 eyes (94.6%) demonstrated a negative FoBMO axis angle (fovea below the ONH). Forty nine eyes (22.1%) demonstrated a FoBMO angle more negative than −10°.

### FoBMO axis angle and distance intra and inter-delineator variability (Figure 5)

**Figure 5:**
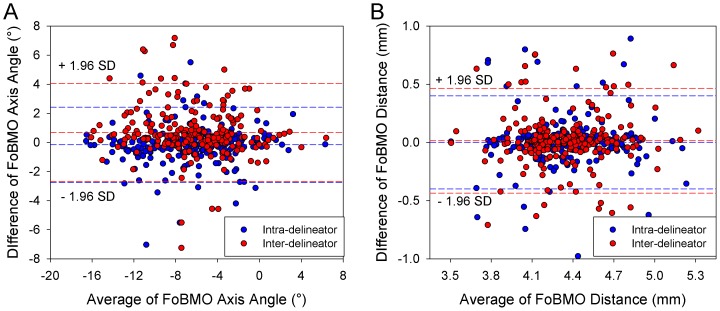
Bland-Altman plots of intra-observer and inter-observer agreement for FoBMO axis angle (A) and FoBMO distance (B). For FoBMO axis angle, intra-delineator limits of agreement (LOA) was −2.8° to 2.4° while inter-delineator LOA was −2.7° to 4.1°. For FoBMO distance, intra-delineator LOA was −439.2 μm to 467.7 μm while inter-delineator LOA was −402.0 μm to 402.7 μm.

Bland-Altman plots of intra- and inter-delineator variability in FoBMO axis and distance are presented in Figure 5. FoBMO axis angle intra-delineator limits-of-agreement were −2.8° to 2.4°. FoBMO axis angle inter-delineator limits of agreement were −2.7° to 4.1° FoBMO distance intra- and inter-delineator limits-of-agreement were −439.2 μm to 467.7 μm and −402.0 μm to 402.7 μm, respectively.

Median interclass correlation coefficients (ICC) for intra-delineator and inter-delineator FoBMO axis angle were 0.949 and 0.899 (Table 2). Median ICC for intra-delineator and inter-delineator FoBMO distance were 0.788 and 0.738.

**Table 2:** Intra- and inter-delineator agreement for FoBMO axis angle and distance.

		FoBMO Axis Angle		FoBMO Distance	
		Intra-delineator	Inter-delineator	Intra-delineator	Inter-delineator
ICC	Median	0.949	0.899	0.788	0.738
	95% CI	0.934–0.961	0.871–0.922	0.733–0.833	0.671–0.792
P-value	Paired t-test	0.060	**<0.001**	0.979	0.356

### Eye-Specific neuroretinal rim gradient by FoBMO sectors (Figure 6)

**Figure 6:**
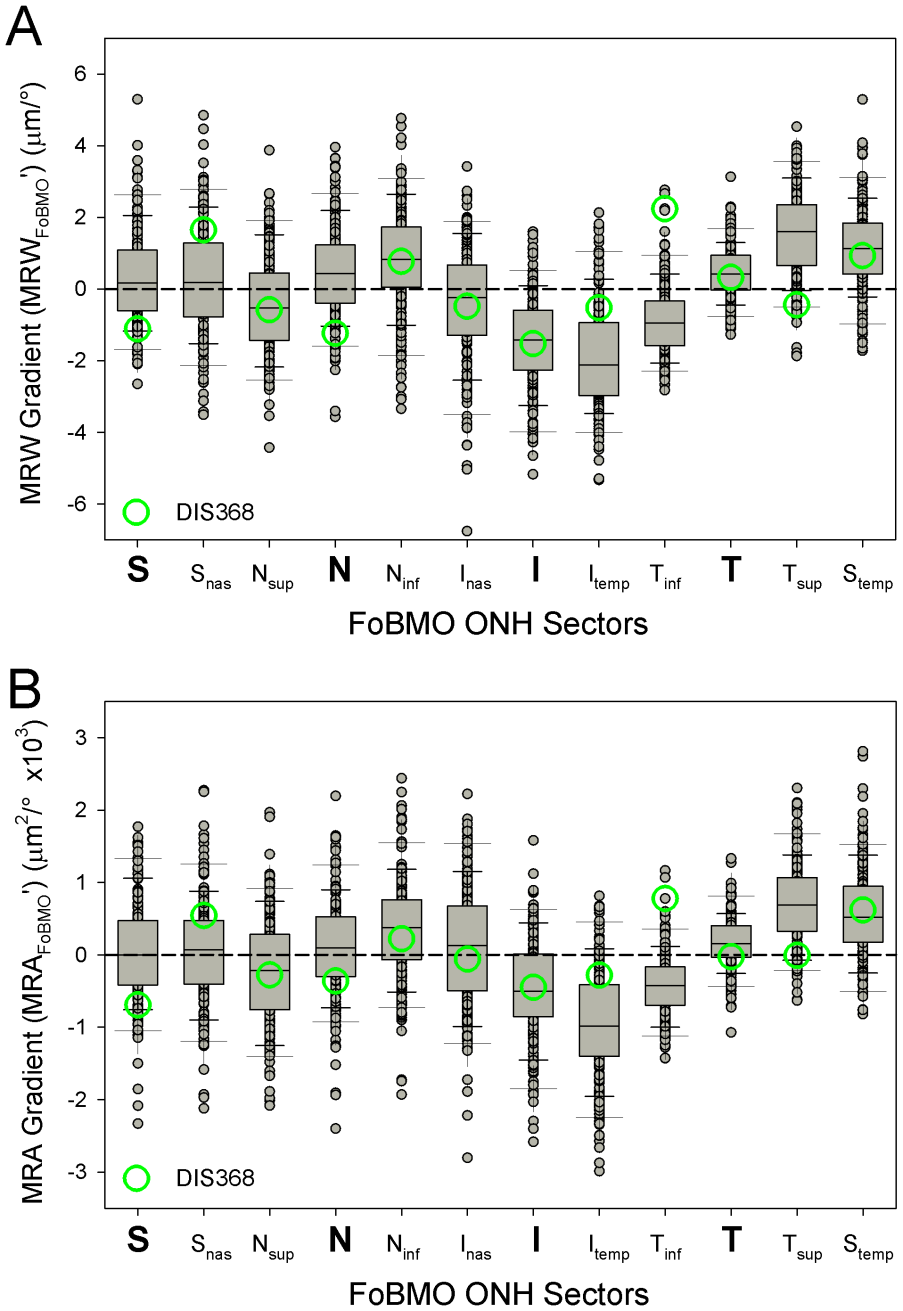
SDOCT MRW (upper) and MRA (lower) gradient in actual values by FoBMO 30° sector for all 222 eyes. Median sectoral MRW gradient was largest in the I_temp_ (−2.1 μm/°) and T_sup_ (1.6 μm/°) sectors. Median sectoral MRA gradient was also largest in the I_temp_ (−988 μm^2^/°) and T_sup_ (689 μm^2^/°) sectors.

Within these 222 high-risk ocular hypertensive and glaucoma eyes, median sectoral MRW gradient was largest in the I_temp_ (−2.1 μm/°) and T_sup_ (1.6 μm/°) sectors (Figure 2C). The absolute value of eye-specific, sectoral MRW and MRA gradients ranged from −6.8 to 5.3 μm/° and −2991 to 2811 μm^2^/° respectively. The inferior and temporal sectors (right half of Figure 6A and 6B) demonstrated larger gradients compared to the nasal and superior sectors (left half of Figures 6A and 6B) for both MRW and MRA.

### FoBMO vs. AIF 30° sectoral rim discordance (Figures 7 and 8)

**Figure 7:**
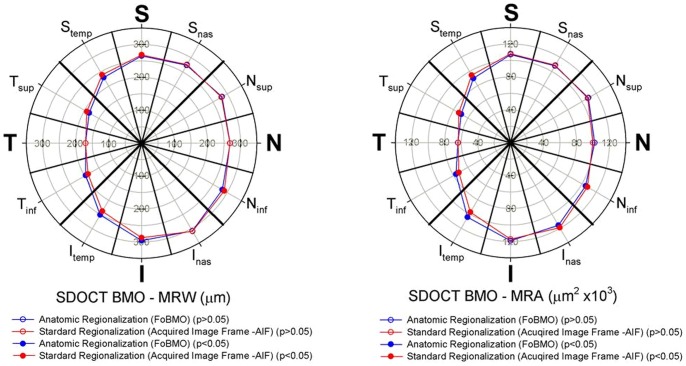
Mean sectoral MRW (left) and MRA (right) for FoBMO regionalization (red) and AIF regionalization (blue) for all 222 study eyes. Wilcoxon signed rank test with Bonferroni correction was applied to compare the two means in the twelve sectors. Sectors of which differences were statistically significant (p<0.05) were shown in solid symbols while sectors of which differences were not statistically significant (p>0.05) were shown in open symbols. These data suggest that when reported as the means of all 222 eyes, the sectoral discordance between AIF and FoBMO regionalization is statistically significant but small. (See Supplemental Table S1 for the sectoral mean values and p values).

**Figure 8:**
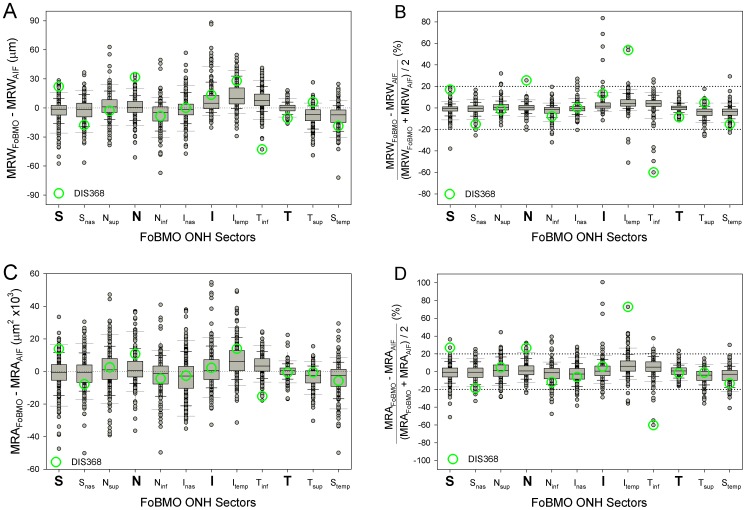
Distribution of Eye-specific, AIF vs. FoBMO discordance for MRW (above) and MRA (below) by 30° sector in actual values (left) and percentage (right). At each sector, the long whiskers correspond to the lowest 5% and upper 95% of the distribution while the short whiskers correspond to the lowest10% and upper 90%, respectively. The bars with the separator in the middle correspond to lower quartiles (25%), upper quartiles (75%) and medians (50%). The percentage difference in most eyes for both MRW and MRA are within ±20% (horizontal dotted lines). However, in some eyes, the difference can be up to 80%. Eye DIS368, (green circles – and also seen in Figures 1 and 2), has three sectors with a difference of more than 20% for MRW and four for MRA. These data suggest that while the overall mean differences reported in Figure 7 can be small, eye-specific discordance within sectors for a subset of eyes is substantial.

The difference between the sectoral mean MRW (n = 222 eyes) calculated on the basis of FoBMO vs. AIF regionalization varied by sector (Figure 7, left) but were generally small, ranging from 0 to 13 μm (Supplemental Table S1) but achieved statistical significance in 7 of 12 sectors (Wilcoxon non-parametric test with Bonferroni correction, p<0.05). FoBMO vs. AIF differences in sectoral mean MRA values for each sector (Figure 8, right), were also small ranging from 268 to 7052 μm^2^ (Supplemental Table S1) but achieved statistical significance in 6 of 12 segments (Wilcoxon signed-rank test with Bonferroni correction, p<0.05) (Figure 8B). Five sectors demonstrated significant FoBMO vs. AIF differences for both MRW and MRA sectoral means (N_inf_, I_temp_, T_inf_,T_sup_ and S_temp_ sectors – Supplemental Table S1). FoBMO vs. AIF discordance in the sectoral means of both MRW and MRA occurred in the I_temp_ sector (Figure 7).

**Figure 9:**
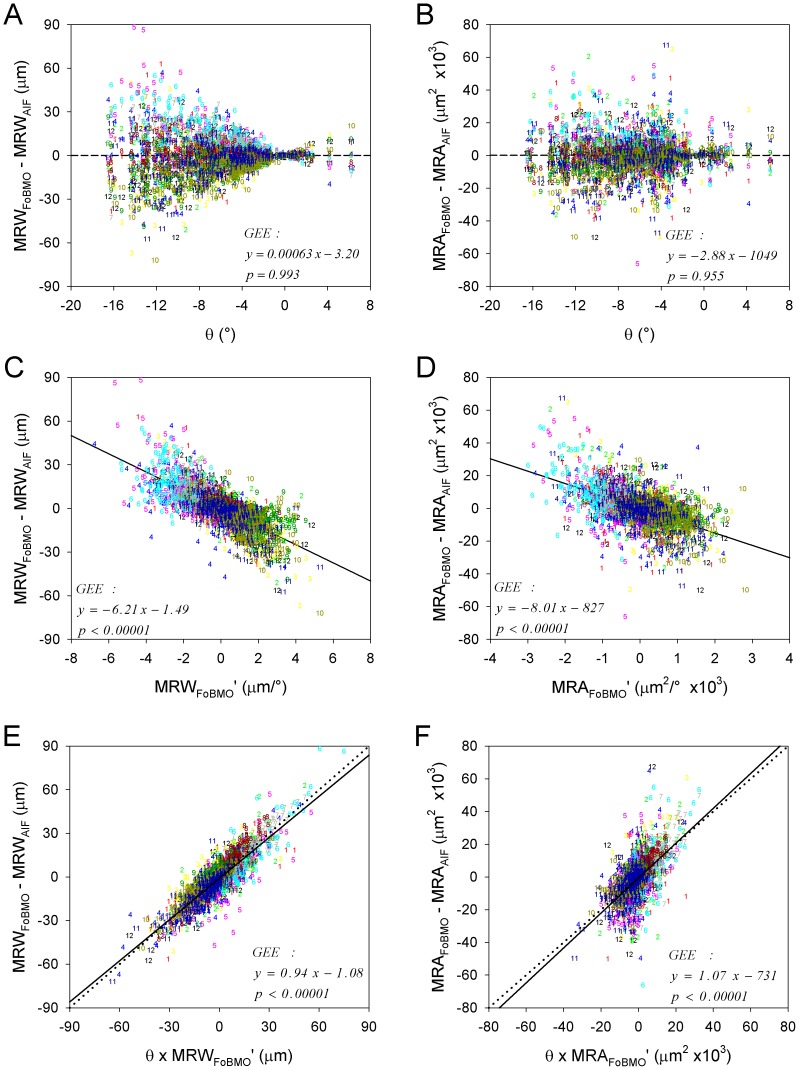
Sectoral FoBMO vs. AIF discordance for MRW (left) and MRA (right) predicted by a univariate generalized estimating equation (GEE) model using FoBMO angle (A & B), one-sided rim gradient (C & D) (see methods) and the interaction of both (E & F). The color coded numbers (1–12) correspond to the FoBMO 30° sectors of each study eye in right eye configuration (Figure 8A and B) expressed as clock-hours (12 - superior, 3 - nasal, 6 - inferior and 9 – temporal, see Figure 2). Eye-specific sectoral AIF vs. FoBMO discordance for both MRW and MRA is better predicted by a combination of FoBMO angle (θ) and one-sided sectoral rim gradient (E & F) than by either, alone. Qualitative inspection of these data suggests that sectors from the inferior and superior quadrants commonly demonstrate the highest sectoral AIF versus FoBMO discordance for both MRW and MRA.

While the differences in the overall mean sectoral values were small, eye-specific discordance was more substantial (Figure 8). Eye-specific sectoral discordance between FoBMO-based and AIF-based derivations of MRW exceeded ±20% in at least 1 sector in 24 eyes (10.8%), exceeded ±20% in at least 2 sectors in 11 eyes (4.9%) and exceeded ±20% in at least 3 sectors in 6 eyes (2.7%) (data not shown). Eye-specific discordance for MRA (Figure 8, lower) exceeded ±20% in at least 1 sector in 87 eyes (39.1%), in at least 2 sectors in 38 eyes (17.1%) and in at least 3 sectors in 27 eyes (12.2%) (data not shown). Qualitative inspection of sectoral, eye-specific discordance (Figure 9) suggests that most of the eyes exceeding ±20% difference were in the I, I_temp_ and the T_inf_ sectors, which are the same sectors that demonstrated the largest rim gradients.

### Factors affecting the magnitude of eye-specific FoBMO vs. AIF rim parameter discordance (Figure 9)

The results of GEE modeling to predict eye-specific FoBMO vs. AIF MRW discordance are shown in Table 3, and illustrated in Figure 9. Three models were tested: 1) FoBMO angle (θ) alone; 2) rim gradient (*MRW_FoBMO_*') alone; and 3) their interaction (*θ × MRW_FoBMO_*'). Figure 9E shows that the interaction term *θ × MRW_FoBMO_*' best predicted FoBMO vs. AIF MRW discordance and yielded a slope coefficient close to unity, expressed as *0.94 × (θ × MRW_FoBMO_*'*)−1.08*. Using the same definitions, the best fit model for MRA discordance was *1.07 × (θ × MRA_FoBMO_*'*)−731* (Figure 9F). The coefficients and corresponding p-values for MRA discordance are also listed in Table 3. Taken together, these data suggest that sectoral FoBMO vs. AIF MRW and MRA discordance is highest in sectors with high rim gradients from eyes with large FoBMO angles.

**Table 3:** Generalized estimating equation (GEE) slope coefficients (b_1_) and p-values for the prediction of sectoral AIF vs. FoBMO sectoral discordance using FoBMO angle and a one-sided definition of rim gradient (Figure 7).

	MRW_FoBMO_ - MRW_AIF_		MRA_FoBMO_ - MRA_AIF_	
	*b_1_*	*p*	*b_1_*	*p*
FoBMO angle (*θ*)	−0.00063	0.993	−2.88	0.955
Rim Gradient (*MR_FoBMO_*')	−6.21	<0.00001	−8.01	<0.00001
*θ × MR_FoBMO_*'	0.94	<0.00001	1.07	<0.00001

After accounting for FoBMO angle and rim gradient (as outlined above) sectoral differences were found to independently contribute to AIF vs. FoBMO MRW and MRA discordance (MRW: χ^2^ = 742, p<0.001; MRA: χ^2^ = 314, p<0.001).

## Discussion

Ideally, a reference axis for regionalization of ONH and retinal structural measurements should be based on meaningful anatomical features that are also readily detectable by clinical imaging, stable over time and consistent between eyes. The reason to use the FoBMO axis to standardize ONH regionalization in all human eyes is to minimize the intra- and inter-individual variation caused by the combined effects of cyclotorsion and/or anatomic differences in the relationship between the ONH and fovea. When the head tilts, the AIF axis changes its position relative to the tissues of the ONH, RNFL and macula. The FoBMO angle changes, (because the AIF axis has changed), but the relation of the FoBMO axis relative to the fovea, RNFL and ONH remains constant. It is thus reasonable to predict that the confidence intervals of reference databases that are regionalized relative to the FoBMO axis should tighten (improving diagnostic performance) and that structure/structure correlations in normal and glaucomatous eyes should be enhanced.

This study expands upon previous descriptions of the importance of the anatomic relationship between the position of the optic disc and fovea and the axis connecting the two [9], [11], [13], [14], [19]. Herein we report descriptive statistics of the FoBMO axis angle and distance within SDOCT images from a population of 222 eyes of 222 participants with high-risk ocular hypertension and glaucoma. To do so we retrospectively identified the fovea within the infrared image associated with SDOCT ONH datasets in which BMO had been hand-delineated. We then characterized eye-specific ONH neuroretinal rim gradient within each eye. Finally, we quantified the magnitude of 30° sectoral discordance in FoBMO versus AIF regionalization of SDOCT MRW and MRA data, and sought predictive factors influencing their magnitude and location. The principal findings of these studies are as follows.

First, while the median FoBMO angle in these eyes was −6.6°, the range encompassed 24° (from −17° to +7°) which is almost equivalent to one full 30° sector (i.e. one clock-hour). Second, the gradient of rim width (and subsequent derivation to rim area) varied by sector, being greatest in the inferior-temporal and temporal-superior sectors. Third, though the differences between FoBMO and AIF-based measurements of ONH rim tissue were generally small for the population averages in each of the 30° sectors, the variation in the magnitude of eye specific discordance among the 222 eyes was substantial with 10.8% of the eyes demonstrating a 20% difference in FoBMO vs. AIF MRW in at least one sector and 39.2% demonstrating a similar difference in MRA. Fourth, in general sectors from the inferior and superior quadrants demonstrated the highest FoBMO vs. AIF discordance. Fifth, a combination of FoBMO angle and rim gradient best predicted the sectors (and eyes) in which FoBMO vs. AIF rim discordance exceeded 20%.

The median value and range of FoBMO axis and distance that we report are similar to a series of previous studies which made a measurement of the ONH-foveal axis using techniques that were different than ours [9], [11], [13], [14], [19]. Initial studies of the angle and distance between the fovea and the center of the ONH have been based on the clinical disc margin within a clinical photograph [19], [30], [31], or SLO reflectance image [5], [9], [32]. Patel et al[14] used the SDOCT-determined neural canal opening [33]–[35] (previous terminology that is equivalent to BMO) and fovea to co-localize RNFLT measurements from two different SDOCT instruments. However in this study, the range of the FoBMO angle relative to the AIF was not a primary outcome and was not reported.

The FoBMO axis angle in the current study was retrospectively determined using BMO as delineated within SDOCT ONH radial B-scans and by clinical (2D) identification of the fovea within the corresponding IR CSLO image. Despite these methodological differences, the range of the FoBMO angle observed here among 222 eyes is similar to the range of the fovea-to-disc angle (relative to the AIF) within the current Heidelberg Spectralis RNFL normative database (mean −6°, range −15° to +3°) [36] as well as the range of an anatomically determined FoBMO angle (−17° to +2°) within a new Heidelberg Spectralis ONH normative database that is based on 246 eyes of 246 normal Caucasian participants (personal communication from Dr. Gerhard Zinser, President of Heidelberg Engineering GmBH, Heidelberg, Germany).

Though the concept and clinical importance of focal and/or regional rim loss in glaucoma has been well known for many years, to our knowledge, eye-specific, sectoral SDOCT rim gradient has not previously been quantitatively characterized. In this study we defined 30°sectoral rim gradient to be the difference in rim width or rim area relative to its neighboring sectors. The purpose of eye-specific rim gradient quantification in this report was to assess its contribution to AIF vs. FoBMO rim discordance. The one-sided approach was chosen because it gave the best prediction of FoBMO vs. AIF discordance. While other strategies for rim gradient quantification such as a two-sided or absolute percent approach demonstrated prediction slopes that were far from unity, they may better discriminate early glaucoma. In the future, we will quantify the same parameter in a series of normative databases so as to assess its potential contribution to the detection of glaucomatous damage in eyes that either have early glaucomatous visual field loss or are clinically determined to be at risk for its development.

The fact that the discordance between sectoral ONH rim parameters derived using the FoBMO reference frame versus the AIF was substantial for individual eyes but not for the population average value has clinical importance. Prior studies have found that study population average values for ONH rim measurements made within stereophotos [32] or confocal scanning laser tomography (CSLT) [37] compare favorably with those made by SDOCT. However, eye-specific discordance revealed a substantial number of eyes in which differences were large, though the magnitude and opposing polarity of these differences averaged close to zero. In our study, the small FoBMO vs. AIF differences for population average values similarly mask the important clinical finding that eye-specific differences can be substantial.

The fact that sectors from the inferior and superior quadrants demonstrated the highest FoBMO vs. AIF discordance (Figure 9) is important because numerous studies have shown that onset and progression of glaucomatous ONH damage most commonly occurs within these sectors [38]. Our study eyes represent a spectrum of glaucomatous damage that ranges from suspicious discs to moderate visual field loss. They also demonstrate the greatest rim gradients within these regions. Our rim gradient data may therefore be a reflection of the location of early to moderate glaucomatous rim change in these eyes. Future studies comparing these eyes to age and population matched normal databases will be necessary to understand the importance of this result.

Our study has the following limitations. SDOCT ONH datasets were not acquired relative to the FoBMO axis, nor was the location of the fovea anatomically determined using SDOCT. The logic for acquiring SDOCT datasets relative to the FoBMO axis has been the subject of a previous report [18]. While that capability has recently been accomplished as part of a separate, SDOCT ONH, peripapillary retinal and macular normative database collection, (a collaborative manuscript is in preparation), it was not available in 2009 and 2010 when this study's SDOCT datasets were acquired. The importance of FoBMO acquisition of SDOCT ONH data remains to be determined and will be the subject of future reports.

We could not perform complete corrections for the transverse magnification of each individual eye in this study because axial length was not measured; only corneal curvatures were entered into the instrument's calculation of transverse magnification. While the FoBMO distance data reported here are thus not completely corrected for eye-specific transverse magnification, the FoBMO axis angle data should not be affected by differences in transverse magnification.

While BMO anatomy was manually delineated within the SDOCT ONH datasets, we did not have macular SDOCT datasets in these eyes from which to identify the fovea geometrically in 3D. Therefore, the fovea was retrospectively identified within the IR CSLO image acquired simultaneously with the SDOCT ONH B-scans. This was done first by a single clinician and the FoBMO axis for each eye was determined using this foveal location and the centroid of the 48 delineated BMO points. This foveal delineation was then repeated by the primary delineator 2 weeks later and by a secondary delineator to characterize the intra and inter-delineator reproducibility. While inter-delineator variation was significantly larger than intra-delineator variation, both the intra-delineator and inter-delineator ICCs were 0.899 or above suggesting that our retrospective approach to identifying the FoBMO axis was reliable.

Given the fact that the 95% CI of the FoBMO axis angle among the 222 eyes spanned nearly 17° and the FoBMO axis angle inter-delineator limits of agreement were −2.7° to 4.1°, FoBMO angle variation caused by foveal delineation may be up to one-third of that caused by actual variation in foveal-ONH anatomy. However, anatomic assignment of the BMO centroid, fovea and FoBMO axis, at the time of SDOCT image acquisition [6], will reduce this component of FoBMO variability in future studies.

AIF vs. FoBMO rim discordance will likely be less important using Garway-Heath ONH sectors [30] because they are larger, ranging from 40 to 110 degrees, and therefore less strongly influenced by focal rim gradient. We believe that FoBMO-based, Garway-Heath regional analysis will continue to be important. However, we also believe that by combining anatomic consistency (the use of the FoBMO axis as the Nasal-Temporal Axis) and eye tracking [39], 30° anatomic precision in regional rim characterization both within and between eyes is now technologically feasible. In correlating to the 12 clock-hours, 30° sectors are clinically intuitive and easy to visualize [18]. Their size allows for a “focal” rather than a “regional” rim gradient characterization. While reproducibility studies are necessary and underway, future studies to assess the performance of 30° and sub-30° FoBMO regionalization in the discrimination of glaucoma, its progression and the enhancement of structure/structure and structure/function relations are also warranted.

In summary, we quantitatively characterized both the FoBMO axis and eye-specific ONH sectoral rim gradient in high risk ocular hypertensive and glaucomatous human eyes so as to characterize their effect on FoBMO versus AIF discordance in SDOCT rim assessment. We found that 10.8% (using SDOCT MRW) and 39.2% (using SDOCT MRA) of the 222 studied eyes demonstrated a 20% difference in AIF vs. FoBMO values in at least one 30°sector. We also found that this occurred most commonly in sectors from the inferior and superior quadrants and specifically within sectors with the greatest sectoral rim gradient and/or eyes with the greatest FoBMO angle. Studies to assess the effects of using FoBMO vs. AIF regionalization and sectoral rim gradient in the discrimination of glaucoma suspect, glaucoma and normal eyes are underway.

## Supporting Information

Table S1 **Mean and SD of sectoral MRW and MRA using FoBMO axis and AIF horizontal axis and their differences.** Wilcoxon signed-rank test with Bonferroni correction was applied to compare the means and sectoral calculated p-values are listed.
(DOCX)
