# Supplementary material for: Anatomic vs. Acquired Image Frame Discordance in Spectral Domain Optical Coherence Tomography Minimum Rim Measurements
Source: PLoS One. 2014 Mar 18;9(3):e92225. doi: 10.1371/journal.pone.0092225 (PMC3958478; doi:10.1371/journal.pone.0092225)
Supplement: Table S1 — Mean and SD of sectoral MRW and MRA using FoBMO axis and AIF horizontal axis and their differences. Wilcoxon signed-rank test with Bonferroni correction was applied to compare the means and sectoral calculated p-values are listed. (DOCX) [file pone.0092225.s001.docx]

**Supplemental Table S1.**

**Mean and SD of sectoral MRW and MRA using FoBMO axis and AIF horizontal axis and their differences. Wilcoxon signed-rank test with Bonferroni correction was applied to compare the means and sectoral calculated p-values are listed**.

|  | MRW (μm) | | | | | | | MRA (μm^2^) | | | | | | |
| --- | --- | --- | --- | --- | --- | --- | --- | --- | --- | --- | --- | --- | --- | --- |
| Sector | FoBMO | | AIF | | FoBMO - AIF | | P-value | FoBMO | | AIF | | FoBMO - AIF | | P-value |
|  | Mean | SD | Mean | SD | Mean | SD |  | Mean | SD | Mean | SD | Mean | SD |  |
| S | 265 | 80 | 268 | 82 | -3.2 | 12.3 | **0.003** | 106973 | 33409 | 108003 | 33254 | -1030 | 11676 | 1.000 |
| S_nas_ | 273 | 84 | 276 | 85 | -2.5 | 12.9 | 0.086 | 107733 | 33497 | 108627 | 33317 | -894 | 10541 | 1.000 |
| N_sup_ | 282 | 90 | 279 | 91 | 2.1 | 13.0 | 0.183 | 109061 | 34564 | 108068 | 35651 | 993 | 12823 | 1.000 |
| N | 269 | 89 | 268 | 88 | 0.6 | 11.3 | 1.000 | 102004 | 34603 | 99837 | 32572 | 2167 | 11163 | 0.332 |
| N_inf_ | 283 | 86 | 289 | 87 | -6.7 | 14.0 | **<0.001** | 105166 | 32430 | 107591 | 34411 | -2425 | 13187 | **0.019** |
| I_nas_ | 308 | 90 | 309 | 90 | -1.1 | 13.7 | 0.718 | 115931 | 35103 | 119122 | 35689 | -3190 | 12820 | **<0.001** |
| I | 296 | 93 | 287 | 94 | 8.5 | 15.5 | **<0.001** | 119066 | 40705 | 117173 | 39852 | 1894 | 13042 | 0.523 |
| I_temp_ | 251 | 86 | 238 | 81 | 12.8 | 13.6 | **<0.001** | 104521 | 38337 | 97469 | 35323 | 7052 | 11251 | **<0.001** |
| T_inf_ | 196 | 58 | 188 | 54 | 8.0 | 11.7 | **<0.001** | 76454 | 24778 | 72518 | 23801 | 3936 | 6688 | **<0.001** |
| T | 170 | 48 | 170 | 47 | 0.0 | 5.3 | 1.000 | 63906 | 19670 | 63639 | 19663 | 268 | 4437 | 1.000 |
| T_sup_ | 184 | 51 | 192 | 54 | -8.2 | 9.8 | **<0.001** | 69086 | 20719 | 72477 | 21543 | -3391 | 6124 | **<0.001** |
| S_temp_ | 230 | 70 | 239 | 74 | -9.4 | 11.9 | **<0.001** | 90192 | 29563 | 94559 | 31018 | -4367 | 10165 | **<0.001** |
|  |  |  |  |  |  |  |  |  |  |  |  |  |  |  |
